# Structure-Inherent Tumor-Targeted IR-783 for Near-Infrared Fluorescence-Guided Photothermal Therapy

**DOI:** 10.3390/ijms25105309

**Published:** 2024-05-13

**Authors:** Yoonbin Park, Min Ho Park, Hoon Hyun

**Affiliations:** 1 Department of Biomedical Sciences, Chonnam National University Medical School, Hwasun 58128, Republic of Korea; unb1n0213@naver.com; 2 BioMedical Sciences Graduate Program (BMSGP), Chonnam National University, Hwasun 58128, Republic of Korea; 3 Department of Surgery, Chonnam National University Medical School and Hwasun Hospital, Hwasun 58128, Republic of Korea

**Keywords:** IR-783, heptamethine cyanine dyes, photothermal therapy, near-infrared fluorescence imaging, tumor targeting

## Abstract

IR-783, a commercially available near-infrared (NIR) heptamethine cyanine dye, has been used for selective tumor imaging in breast, prostate, cervical, and brain cancers in vitro and in vivo. Although the molecular mechanism behind the structure-inherent tumor targeting of IR-783 has not been well-demonstrated, IR-783 has unique properties such as a good water solubility and low cytotoxicity compared with other commercial heptamethine cyanine dyes. The goal of this study is to evaluate the phototherapeutic efficacy of IR-783 as a tumor-targeted photothermal agent in human colorectal cancer xenografts. The results demonstrate that IR-783 shows both the subcellular localization in HT-29 cancer cells and preferential accumulation in HT-29 xenografted tumors 24 h after its intravenous administration. Furthermore, the IR-783 dye reveals the superior capability to convert NIR light into heat energy under 808 nm NIR laser irradiation in vitro and in vivo, thereby inducing cancer cell death. Taken together, these findings suggest that water-soluble anionic IR-783 can be used as a bifunctional phototherapeutic agent for the targeted imaging and photothermal therapy (PTT) of colorectal cancer. Therefore, this work provides a simple and effective approach to develop biocompatible, hydrophilic, and tumor-targetable PTT agents for targeted cancer phototherapy.

## 1. Introduction

Photothermal therapy (PTT) is emerging as a safe, effective, noninvasive, and inexpensive modality for treating various types of cancer, compared to traditional treatment approaches such as surgery, chemotherapy, and radiotherapy. Basically, PTT relies on photothermal transduction agents for converting light energy into local hyperthermia, leading to cancer cell death [1,2]. When photons radiated by a near-infrared (NIR) method collide with small-molecule chromophores, electrons are excited from their ground state (S_0_) to a higher singlet state (S_1_). Then, they are relaxed to the lowest excited state through internal conversion. The relaxed molecules at the lowest vibrational level of the excited state can decay back to the ground state (heat generation) following the non-radiative relaxation path. The photothermal effects are generally the result of the attenuation of non-radiative relaxation paths [3]. Currently, most PTT methods have focused mainly on the laser wavelength in the NIR range (650–1700 nm) with a low power density for a short irradiation time, because the NIR light has a deeper tissue penetration and causes less damage compared with a shorter wavelength light to tissues [4,5,6]. To achieve an accurate, efficient, and reliable phototherapeutic effect against cancer, the sufficient tumor accumulation of PTT agents is the key for the high efficacy of PTT and minimal damage to nearby healthy tissue. Many different types of PTT agents, including organic, metal nanomaterials, and polymethine cyanine dyes with a strong NIR extinction coefficient and high photothermal conversion efficiency, have been continually developed [7,8,9]. Nevertheless, high levels of tumor accumulation of the various nanomaterials without systemic toxicity is a particularly important issue for design considerations related to complicated synthetic processes and unsolved biological safety [10]. In this regard, functional cyanine dyes may be an alternative for effective cancer treatment to achieve simultaneous tumor targeting, imaging, and phototherapy.

Previously, commercial heptamethine cyanine dyes such as IR-780, IR-783, IR-786, and IR-808 (also called MHI-148) have been used for tumor-targeted NIR fluorescence imaging through the structure-inherent targeting capacity as a single small molecule without the additional conjugation of tumor-targeting ligands [11,12,13]. Although the tumor-targeting mechanism of the heptamethine cyanine dyes is yet to be fully understood, the meso-chloride on a cyclohexenyl ring of the heptamethine backbone may play a key role in the tumor-specific uptake and retention [14,15]. Usama et al. suggested that the noncovalent or covalent albumin adducts, which can be formed between plasma albumin and the heptamethine cyanine dyes after injection into the bloodstream, were effectively trapped in tumors through the receptor-mediated endocytosis of albumin [16,17]. Otherwise, organic anion transporting polypeptides (OATPs), which are overexpressed at the plasma membrane of cancer cells, are well-recognized determinants of tumor uptake to explain the structure-inherent targeting mechanism of the heptamethine cyanine dyes [18].

Among the tumor-targeted cyanine dyes commercially available, IR-783 is a representative heptamethine cyanine dye with a good water solubility, owing to its charged structure having two sulfonate groups. Until now, IR-783 has been used continually for tumor imaging in breast, prostate, cervical, and brain cancers in vitro and in vivo, owing to its tumor-selective targeting activity [19,20,21,22]. In particular, IR-783 exhibits a good biocompatibility and less uptake in the reticular and endothelium of the liver or spleen, compared with that of the FDA-approved NIR dye indocyanine green (ICG) showing a high liver uptake [23,24]. Unlike the ICG structure, the chlorine atom on the bridging ring of IR-783 can be reactive towards a variety of nucleophiles, thereby enabling the conjugation of various anticancer drugs for targeted chemotherapy. Due to the high tumor uptake but low cytotoxicity of IR-783, Guan et al. investigated the anticancer efficacy of the IR-783/genistein conjugate in MCF-7 tumor xenografts of a mice breast cancer model, which displayed improved chemotherapeutic properties compared with the genistein alone [25]. Moreover, Huang et al. demonstrated that the IR-783/farnesylthiosalicylic acid conjugate exhibited a superior tumor targetability and anticancer therapeutic efficacy against six human breast cancer cell lines compared with the farnesylthiosalicylic acid alone both in vitro and in vivo [20]. Interestingly, there are no reports of phototherapeutic applications using the IR-783 alone as a single small molecule without the incorporation of nanomaterials. The combination of tumor-targeted IR-783 and NIR laser irradiation may become a simple and powerful tool for potential clinical use.

In this study, we aimed to investigate the photothermal therapeutic effect of IR-783 combined with an 808 nm laser on colorectal cancer cells in vitro and in vivo. Moreover, we emphasize that this is the first report to confirm the tumor-targeting efficiency of IR-783 in vivo using a HT-29 human colorectal cancer cell line. Our results suggested that IR-783 not only showed a high tumor uptake in a HT-29 xenograft mouse model but also revealed an excellent heat generation capability under the 808 nm laser irradiation for effective photothermal cancer treatment. Based on the concept of structure-inherent targeting, the water-soluble IR-783 was gradually accumulated in the tumor site 24 h post-injection, which was the optimal timing of the laser irradiation to maximize the light-to-heat conversion efficiency for noninvasive tumor ablation. Therefore, this work provides a simple and prospective strategy for the design of tumor-targeted cyanine dyes used for NIR fluorescence-guided cancer phototherapy.

## 2. Results

### 2.1. Optical and Structural Characterization of IR-783

**Figure 1:**
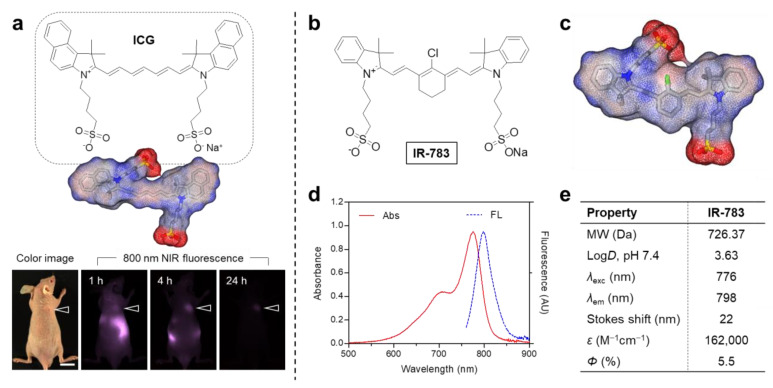
(**a**) Chemical structure and tumor-targeting efficiency of ICG. The tumor sites are indicated by arrowheads. Scale bar = 1 cm. (**b**) Chemical structure and (**c**) 3D modeling of IR-783. Red, negative charge; blue, positive charge; gray, hydrophobicity. (**d**) Absorption and fluorescence emission spectra of IR-783 measured in PBS at pH 7.4. (**e**) Physicochemical and optical properties of IR-783. In silico calculations of the distribution coefficient (log*D* at pH 7.4) and surface charge density distribution were performed using Marvin and JChem calculator plugins (ChemAxon).

Since the chemical structure of IR-783 is similar to ICG, we first reconfirmed the tumor-targeting efficiency of the clinically available ICG, as reported previously [26]. As expected, ICG exhibited no significant uptake in the tumor tissue within 24 h of injection (Figure 1a). As shown in Figure 1b, IR-783 is composed of a chloro-cyclohexenyl ring on the heptamethine skeleton, unlike that of ICG, and two sulfonate groups on each side chain. It is well-known that the charged groups, sulfonate groups frequently found within the commercial cyanine dyes, are clearly hydrophilic and, thus, tend to improve water solubility. Additionally, the most important difference between IR-783 and ICG is the presence of the meso-chloride on a cyclohexenyl ring of the heptamethine chain, which could affect the tumor-specific accumulation by the formation of albumin adducts, as reported previously [27]. This is also supported by the 3D modeling of geometrical positions of IR-783 and ICG, which are twisted in the same direction (Figure 1c). The absorption and fluorescence emission spectra of IR-783 were measured in phosphate-buffered saline (PBS, pH 7.4), owing to its good water solubility (Figure 1d). As summarized in Figure 1e, the peak absorption at 776 nm and maximum fluorescence emission at 798 nm were determined with a typical Stokes shift of 22 nm, respectively. IR-783 displayed moderate molar extinction coefficient (*ε* = 162,000 M^−1^cm^−1^) and quantum yield (*Φ* = 5.5%).

### 2.2. In Vitro Cytotoxicity, Cell Binding, and Photothermal Effect

**Figure 2:**
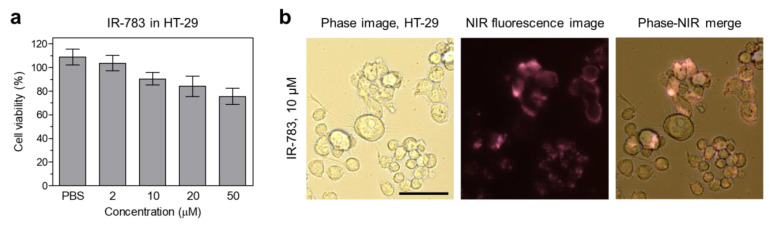
(**a**) Cell viability assay of IR-783 using HT-29 cancer cells. Percentage cytotoxicity is determined after 4 h of treatment with various concentrations of IR-783. (**b**) Live-cell binding of IR-783 in HT-29 cancer cells. Phase contrast and NIR fluorescence images are obtained after 4 h of incubation with 2 μM of IR-783. Images are representative of n = 3 independent experiments. All fluorescence images had identical exposure times and normalization. Scale bars = 100 μm.

The in vitro cytotoxicity and cellular uptake of IR-783 were performed using the 3-(4,5-dimethylthiazol-2-yl)-2,5-diphenyltetrazolium bromide (MTT) assay in HT-29 cancer cells after 4 h of incubation with IR-783 at various concentrations (2–50 μM) (Figure 2a). As expected, IR-783 showed no significant cytotoxicity to the HT-29 cancer cells even at the high concentration of 50 μM. This result is consistent with previous reports and indicates that the IR-783 itself has no therapeutic efficacy without NIR laser irradiation. Additionally, we confirmed the cellular uptake of IR-783 after 4 h of incubation in HT-29 cells. IR-783 exhibited distinct NIR fluorescence signals in the cell membrane (Figure 2b). This suggests that the photothermal energy generated from IR-783 under NIR laser irradiation can be delivered effectively into the HT-29 cancer cells to induce apoptosis and/or necrosis.

**Figure 3:**
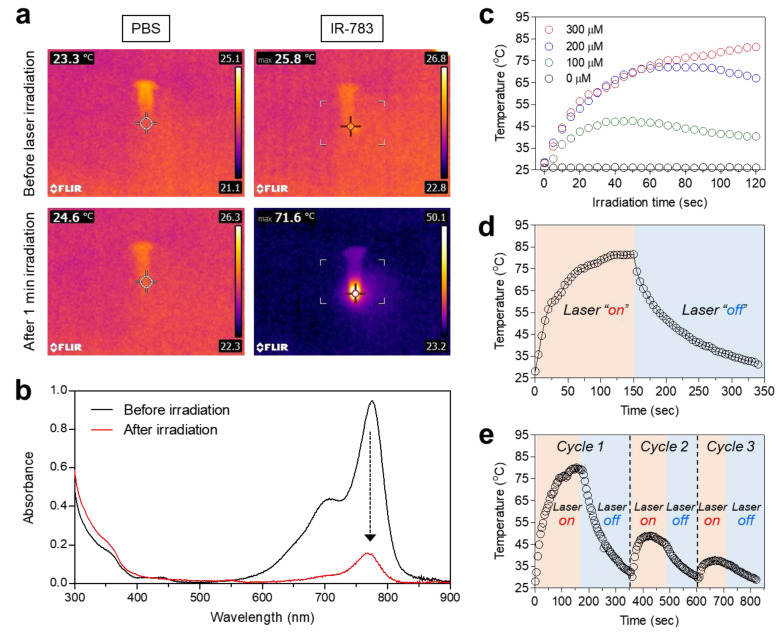
(**a**) In vitro thermal images of PBS and IR-783 (300 μM) solutions irradiated with an 808 nm laser at 1.0 W/cm^2^ power density for 1 min. The infrared thermal imager was used to monitor the maximum temperature in real time. (**b**) Photostability of the IR-783 solution under laser irradiation. The absorbance changes were measured before and after 1 min of laser irradiation. (**c**) Temperature changes in PBS and IR-783 (100, 200, and 300 μM) solutions were observed for 120 s of laser irradiation (808 nm, 1.0 W/cm^2^). (**d**) Heating and cooling curve of IR-783 (300 μM) under laser irradiation (808 nm, 1.0 W/cm^2^). (**e**) Temperature changes of IR-783 (300 μM) during three on/off cycles of laser irradiation (808 nm, 1.0 W/cm^2^).

After confirming the in vitro biocompatibility and the cellular uptake study, we explored the photothermal conversion efficiency of IR-783 through monitoring the temperature variation in a solution of IR-783 (300 μM in PBS) during irradiation with an 808 nm NIR laser (1.0 W/cm^2^) for 1 min. The temperature changes were monitored in real time using a thermal imager. The temperature of the IR-783 solution is rapidly elevated by up to 71.6 °C at 1 min post-irradiation, while the PBS alone showed no temperature change under the same conditions (Figure 3a). To analyze the spectral changes, the absorbance of IR-783 dissolved in PBS was measured before and after the 1 min of laser irradiation, respectively (Figure 3b). As expected, the peak absorbance at 776 nm dramatically decreased after 1 min of laser irradiation. This indicates that the polymethine cyanine structure can easily be destroyed by photobleaching during exposure to concentrated NIR light. The temperature of the IR-783 solutions could be effectively increased for 2 min of laser irradiation in a concentration-dependent manner (Figure 3c). With increasing the concentration from 100 to 300 μM, the temperature changes of the solution reached approximately from 40 to 80 °C upon 808 nm laser irradiation (1.0 W/cm^2^) in 2 min. As shown in Figure 3d, the photothermal conversion efficiency (*η*) of IR-783 was calculated to be 28.9%, which is comparable to that of the heptamethine cyanine dyes reported previously [28,29]. This result suggests that the commercial IR-783 can be applied for in vivo photothermal tumor ablation. Moreover, after three cycles of heating and cooling (150 s heating coupled with 200 s cooling for each cycle), the photothermal stability of the IR-783 solution significantly decreased in the second cycle and was mostly minimized in the third cycle under repeated laser irradiation (Figure 3e). This suggests that the in vivo tumor targeting efficiency of IR-783 is highly important for effective photothermal tumor ablation, owing to the irreversible photodegradation of IR-783.

**Figure 4:**
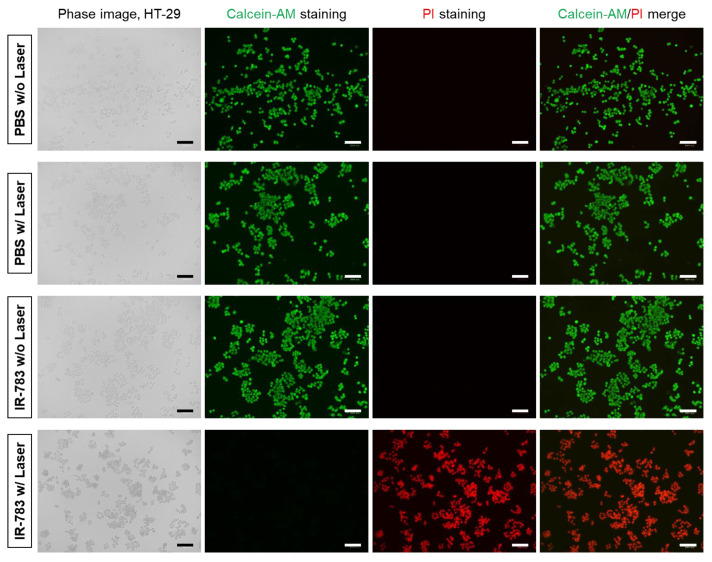
Fluorescence images of HT-29 cells before and after PTT treatment. HT-29 cells were incubated with the 5 µM concentration of IR-783 for 4 h and treated with the 808 nm laser at 1.0 W/cm^2^ for 1 min. HT-29 cells were then costained with calcein-AM (green for live cells) and propidium iodide (PI; red for dead cells). Images are representative of n = 3 independent experiments. All fluorescence images had identical exposure times and normalization. Scale bars = 100 μm.

To check the in vitro cell phototoxicity before and after laser irradiation, HT-29 cancer cells after 4 h of incubation with IR-783 were exposed to 808 nm laser irradiation with the power density of 1.0 W/cm^2^ for 1 min. Compared to the groups of PBS without laser, PBS with laser, and IR-783 without laser irradiation, the treatment group of IR-783 with laser irradiation displayed extensive cell death (Figure 4). The red fluorescent cells stained with propidium iodide (PI) were mainly observed after laser irradiation, whereas the green fluorescent cells stained with Calcein-AM were not detected entirely. Hence, this suggests that the combination of IR-783 and laser irradiation is a promising effective method for photothermal cancer treatment.

### 2.3. Time-Dependent In Vivo Tumor Retention and Photothermal Effect

**Figure 5:**
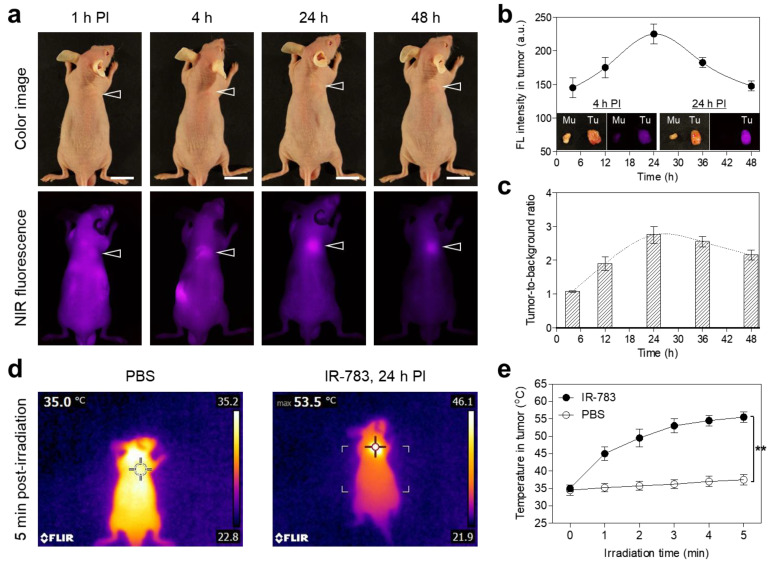
In vivo HT-29 tumor-targeting efficiency of IR-783. (**a**) NIR fluorescence imaging for 48 h after injection of IR-783. The tumor sites are indicated by arrowheads. Scale bars = 1 cm. (**b**) Time-dependent fluorescence intensity and (**c**) tumor-to-background ratio at the tumor site targeted by IR-783. The inset shows the resected tumors 4 h and 24 h after injection of IR-783. Abbreviations: Mu, muscle; Tu, tumor; PI, post-injection. (**d**) Thermal images and (**e**) temperature changes in tumor-bearing mice at the tumor area 24 h after injection of PBS or IR-783, followed by 808 nm laser irradiation (1.0 W/cm^2^) for 5 min. Images are representative of 3 mice per treatment group. All NIR fluorescence images had identical exposure times and normalization. Data are expressed as mean ± S.D. (n = 3). ** *p* < 0.01.

To investigate the in vivo behavior and tumor-targeting capacity of IR-783, HT-29 tumor-bearing mice were subjected to a single intravenous administration of a saline solution containing IR-783 (0.8 mg/kg). Interestingly, IR-783 revealed a significant tumor accumulation at 24 h after injection (Figure 5a). The fluorescence intensity at the tumor site was gradually elevated by up to 24 h post-injection and continuously decreased after that 24 h of injection (Figure 5b). Additionally, the value of the tumor-to-background ratio also increased over 24 h post-injection and was maintained until 48 h of injection, due to the low background signals in the body (Figure 5c). Considering the effective tumor-targeted imaging and photothermal treatment, the optimal time of PTT to avoid the unnecessary damage of neighboring normal tissues can be determined at 24 h after injection. After successfully confirming the optimal accumulation time of the tumor site, we carried out the in vivo PTT treatment at 24 h post-injection of IR-783. The solutions of IR-783 and PBS alone were intravenously reinjected into the HT-29 tumor-bearing mice. Subsequently, the mice were exposed to the 808 nm NIR laser with a 1.0 W/cm^2^ power density for 5 min. The power density of the 808 nm laser (1.0 W/cm^2^) was previously optimized to avoid unnecessary injury in normal tissue due to the laser power alone [30]. Importantly, the tumor temperature of the IR-783 group exhibited a high temperature change (53.5 °C) after 5 min of exposure to the 808 nm NIR laser, whereas the PBS group displayed no change in the tumor temperature under the same irradiation condition (Figure 5d). The tumor temperature in the IR-783 group increased by 50 °C after 2 min of laser irradiation, and then the temperature in the tumor site was maintained up to approximately 55 °C for the next 3 min of laser irradiation (Figure 5e). This result demonstrates that the tumor temperature treated with the IR-783 and NIR laser is high enough to induce tumor necrosis. Consequently, it is proven that the commercial IR-783 can be used as an effective PTT agent for tumor-targeted imaging and photothermal treatment.

### 2.4. In Vivo Photothermal Therapeutic Efficacy

**Figure 6:**
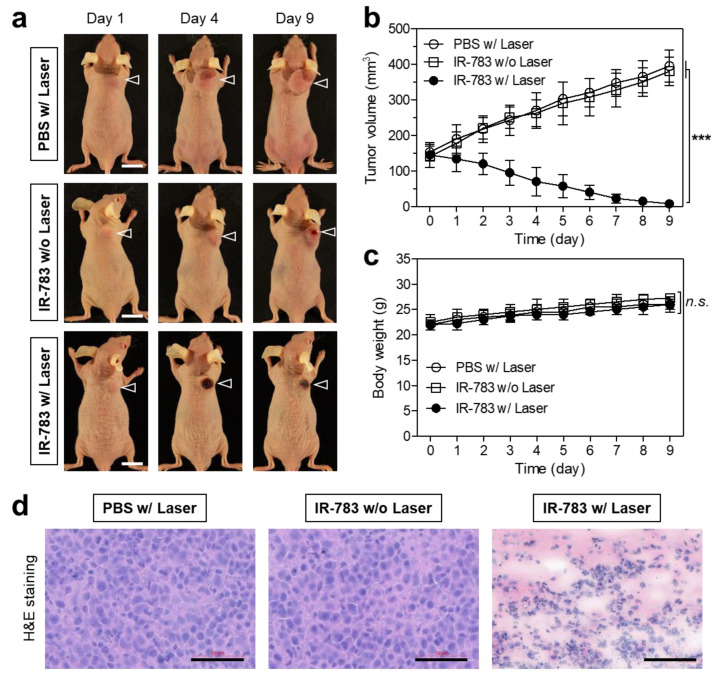
In vivo NIR phototherapeutic efficacy of IR-783. (**a**) Representative photos of changes in tumor size in HT-29 tumor-bearing mice for 9 days after different treatments. The laser groups were treated with 24 h post-injections of PBS or IR-783, followed by 808 nm laser irradiation (1.0 W/cm^2^) for 5 min. Scale bars = 1 cm. (**b**) Tumor growth rates and (**c**) body weights of each treatment group were monitored for 9 days. Data are expressed as mean ± S.D. (n = 3). *** *p* < 0.001. n.s., not significant. (**d**) Histological observation of tumors stained with H&E in each treatment group. Scale bars = 50 μm.

Finally, the efficacy of photothermal tumor therapy under the 808 nm laser irradiation in the HT-29 tumor-bearing mice was evaluated by macroscopic observation of the tumor growth in each treatment group for 9 days (Figure 6a). As expected, in control groups which were treated with PBS with laser irradiation and IR-783 without laser irradiation, the tumor volumes between the two groups displayed no significant difference for 9 days (Figure 6b). This indicates that the laser treatment only or IR-783 alone have no effect on tumor suppression. Most importantly, the tumor group treated with the IR-783 and laser irradiation revealed a significant phototherapeutic effect with complete tumor ablation and no recurrence for 9 days. Therefore, this result suggests that the combination of IR-783 and laser irradiation can induce photothermal cell death in tumors. Moreover, the excellent tumor targetability of IR-783 can contribute to the high efficacy of laser irradiation, thereby conducting safe and accurate PTT. Additionally, no significant loss of body weight in all treatment groups was observed for 9 days of monitoring to evaluate the systemic toxicity of IR-783 (Figure 6c). Furthermore, the tumor tissues collected from each group after 24 h of different treatments were stained with hematoxylin and eosin (H&E) for a histological examination (Figure 6d). The tumor sections treated with PBS with laser irradiation or IR-783 without laser irradiation exhibited typical morphological features of cell proliferation, whereas apparent morphologic alterations such as a decreased number of and shrunken nuclei were observed in the tumor tissues treated with IR-783 with laser irradiation, respectively. This result also demonstrates that the commercial IR-783 could be a safe and biocompatible PTT agent for future clinical applications.

## 3. Discussion

To date, many different types of heptamethine cyanine dyes available commercially have been prepared for the preclinical applications of optical imaging not only in NIR-I (650–900 nm) but also in NIR-II (1000–1700 nm) regions. However, most of them are poorly soluble in aqueous solutions, resulting in limitations of using NIR-emitting fluorescent dyes in biomedical research. Hence, various kinds of nanomaterials such as amphiphilic polymers, albumin, and liposomes have been used to improve the water solubility of the hydrophobic heptamethine cyanine dyes for in vitro and in vivo NIR fluorescence imaging. In this regard, IR-783 is one of the few heptamethine cyanine dyes that have more polarity, thereby increasing the water solubility. Most importantly, IR-783 can be preferentially accumulated in colon tumors through the structure-inherent targeting capacity without conjugation with tumor-targeting ligands, and further applied to photothermal cancer treatment combined with a NIR laser, which we highlight in this study.

Interestingly, the water-soluble IR-783 and ICG are similar with respect to the chemical structures and binding affinity to serum albumin; however, they are very different in the tumor accumulation and retention ability for tumor-specific imaging and treatment. Although ICG has been widely used for image-guided surgery due to its clinical availability and safety, it is well-known that ICG has no tumor-targeting specificity. Thus far, ICG has been predominantly used to evaluate blood flow and the liver clearance capacity in the clinic for over 60 years. To be used for tumor-specific imaging, the complex of ICG with serum albumin has typically been used to enhance the photostability, blood circulation, and tumor-targeting ability of ICG through the enhanced permeation and retention (EPR) effect [31]. Moreover, the tumor-specific uptake of exogenous albumin can be explained by receptor-mediated albumin uptake pathways related to albumin binding proteins such as membrane-associated glycoprotein and secreted protein acidic and rich in cysteine (SPARC) [31]. Therefore, we emphasize that the sulfonated IR-783 similar to ICG can exceptionally accumulate at the tumor site without the use of exogenous albumin or nanomaterials, even though the exogenous albumin mixed with various cyanine dyes before administration can serve as a tumor-targeting carrier, enabling an enhanced tumor accumulation of retention.

Previously, Tian and Bai et al. clarified that the general principle is to prepare both albumin-chaperoned dyes and albumin-escaping dyes by using hydrophobic IR-780 and hydrophilic IR-783 dyes containing the chloro-cyclohexenyl ring on the heptamethine central chain. The authors suggested that the slow binding between the sulfonated IR-783 dye and albumin is considered as a noncovalent combination; thus, the hydrophilic IR-783 dye could be a potential albumin-escaping agent [32,33]. Based on this theory, the tumor-specific accumulation of IR-783 can be supported by the concept of structure-inherent tumor targeting owing to its weak binding affinity to serum albumin. Taken together, it can offer an alternative to image-guided cancer surgery, because IR-783 is the likely replacement for the ICG with respect to the similar chemical structure and low cytotoxicity.

There are several more issues that may need to be addressed in the upcoming study. First, the tumor-targeted imaging of IR-783 should be conducted by using an orthotopic colorectal tumor model, because it is still a challenge to closely monitor and accurately quantify the primary tumor growth, metastatic activity, and response to phototherapy. Second, the nonspecific tissue/organ uptake and slow clearance behaviors of IR-783 are major problems in achieving a sufficient tumor-to-background ratio for accurate tumor-targeted imaging. In our previous study, we first reported the supramolecular complex self-assembled from IR-783 and methyl-β-cyclodextrin to enhance the tumor-specific imaging accompanied by rapid clearance from the body. This strategy may be more applicable to the orthotopic colorectal tumor model. Third, the exact targeting mechanism of IR-783 still remains to be fully identified. A deeper understanding of the tumor-targeting mechanism in the future of cancer treatment may provide new strategies for the design of highly effective cyanine dyes enabling photothermal, photodynamic, and chemodynamic therapy.

In summary, we demonstrated that the water-soluble anionic IR-783 dye can be successfully used for tumor-targeted fluorescence imaging 24 h post-injection in a HT-29 human colon cancer xenograft mouse model and subsequent photothermal cancer treatment under the 808 nm laser irradiation. In the present study, the commercial IR-783 dye showed promise in the detection of colon cancer in vivo through structure-inherent targeting, resulting in a simple and highly effective treatment option for the success of future clinical trials. Furthermore, this work provides a practical strategy to develop biocompatible, hydrophilic, and tumor-targetable PTT agents for targeted cancer phototherapy.

## 4. Materials and Methods

### 4.1. Optical and Physicochemical Property Analyses

IR-783 and ICG were purchased from Sigma-Aldrich (St. Louis, MO, USA) and used as received without further purification. All optical measurements were carried out in PBS at pH 7.4 (Sigma-Aldrich). The absorption spectrum of IR-783 was measured by a fiber optic FLAME spectrometer (Ocean Optics, Dunedin, FL, USA). The molar extinction coefficient (*ε*) of IR-783 was determined by the Beer–Lambert equation. The fluorescence emission spectrum of IR-783 was analyzed using a SPARK^®^ 10M microplate reader (Tecan, Männedorf, Switzerland) at an excitation wavelength of 720 nm and emission wavelengths ranging from 760 to 900 nm. To determine the fluorescence quantum yield (*Φ*) of IR-783, ICG dissolved in dimethyl sulfoxide (DMSO) (*Φ* = 13%) was used as a calibration standard under the conditions of matched absorbance at 770 nm [34]. In silico predictions of the distribution coefficient (log*D* at pH 7.4) and 3D simulation of surface charge density distribution were performed using Marvin and JChem calculator plugins (JChem version 14.12.15.0, ChemAxon, Budapest, Hungary).

### 4.2. In Vitro Live-Cell Imaging

The human colorectal adenocarcinoma cell line (HT-29) was obtained from the American Type Culture Collection (ATCC; Manassas, VA, USA). HT-29 cells were cultured in Roswell Park Memorial Institute (RPMI) 1640 medium (Gibco BRL, Paisley, UK) containing fetal bovine serum (FBS), penicillin, streptomycin, and amphotericin B (Welgene, Gyeongsan, Republic of Korea) on a culture plate. The cultured cells were stored in a humidified incubator set to 5% CO_2_ at 37 °C. Fluorescence microscopic imaging was performed using a 4-filter set of the Nikon Eclipse Ti-U inverted microscope system (Nikon, Seoul, Republic of Korea). Image acquisition and analysis were performed using the NIS-Elements Basic Research software (https://www.microscope.healthcare.nikon.com/products/software/nis-elements/nis-elements-basic-research, accessed on 1 April 2024). All fluorescence images had identical exposure time and normalization.

### 4.3. In Vitro Cytotoxicity Assay

When the HT-29 cells reached a confluence of approximately 50%, cell toxicity and proliferation were evaluated using MTT (Sigma-Aldrich) assay. HT-29 cells were seeded onto 96-well plates (1 × 10^4^ cells per well). To evaluate the cytotoxicity depending on the IR-783 concentration, the cancer cells were treated with IR-783 (2, 10, 20, and 50 μM) for 4 h and cultured for 24 h after treatment. At each time point, the incubation cell medium was replaced with 100 μL of fresh medium, and 10 μL of the MTT solution was directly added to each 100 μL well. Subsequently, the plates were then incubated for 4 h at 37 °C in a humidified 5% CO_2_ incubator. Finally, the plates were placed in a microplate reader (SPARK^®^ 10M, Tecan) to measure the absorption intensity at 570 nm. Cell viability was calculated using the following formula: cell viability (%) = (*A*_sample_ − *A*_blank_)/(*A*_control_ − *A*_blank_) × 100, where *A* is the average absorbance.

### 4.4. In Vitro Photothermal Conversion Efficiency

IR-783 (300 μM) dissolved in PBS (100 μL, pH 7.4) was exposed to laser irradiation at 808 nm (1.0 W/cm^2^). The temperature change was monitored using a thermal imager (FLIR Systems, Wilsonville, OR, USA). The heating and cooling were repeated three times to test the photothermal stability of IR-783. Based on the equations reported previously [35], the photothermal conversion efficiency (*η*) of IR-783 was calculated as follows:

From an energy balance in a system, we can describe the total energy balance, as follows:

(1) $$\underset{i}{\sum }m_{i}C_{p,i}\frac{dT}{dt}=Q_{IR-783}+Q_{s}-Q_{loss}$$

The mass is expressed as *m*. The heat capacity of mixture solvent (water) is expressed as *C_p_*. The solution temperature is expressed as *T*.

The photothermal energy input from the IR-783 is expressed as *Q_IR-783_*. The *Q_IR-783_* is calculated as follows:

*Q_IR_*_-783_ = *I*(1 − 10^−*A*λ^)*η* (2)

The laser power density is expressed as *I*. The absorbance of IR-783 at 808 nm is expressed as *A*_λ_. The photothermal conversion efficiency generated from the absorbed light energy to heat energy is expressed as *η*. The thermal energy associated with the light absorbance of the solvent is expressed as *Q_s_*.

The heat energy lost to the surroundings is expressed as *Q_loss_* and can be calculated as follows:

*Q_loss_* = *hA*Δ*T* (3)

The heat transfer coefficient is expressed as *h*. The surface area of the container is expressed as *A*. The temperature change is expressed as Δ*T*. The Δ*T* is defined as *T* − *T_surr_* (*T*: solution temperature, *T_surr_*: ambient temperature of the surroundings). After turning the light source off, *hA* can be measured by the rate of temperature decrease. Then, the combination of Equations (3) and (1) produces Equation (4):

(4) $$\underset{i}{\sum }m_{i}C_{p,i}\frac{dT}{dt}=-Q_{loss}=-hA\Delta T$$

After rearrangement and integration, the following expression for *t* is obtained as follows:

(5) $$t=-\frac{\sum_{i}m_{i}C_{p,i}}{hA}\theta$$

*θ* is defined as the ratio of Δ*T* to Δ*T*_max_, as follows:

(6) $$\theta =\frac{\Delta T}{\Delta T_{max}}$$

According to the cooling curve, *τ_s_* and heat transfer coefficients (*hA*) can be determined as follows:

*t* = − *τ_s_* ln(*θ*) (7)

At the maximum steady-state temperature, the heat input is equal to the heat output, as follows:

*Q_IR_*_-783_ + *Q_s_* = *Q_loss_* = *hA*Δ*T*_max_ (8)

where Δ*T*_max_ is the temperature change at the maximum steady-state temperature.

Therefore, the photothermal conversion efficiency.of IR-783 can be calculated as follows:

(9) $$\eta =\frac{hA\Delta T_{max}-Q_{s}}{I\left(1-10^{-A\lambda }\right)}$$

### 4.5. In Vitro Photothermal Cytotoxicity

HT-29 cancer cells were incubated with the 5 µM concentration of IR-783 for 4 h. Subsequently, the cells were washed with PBS and treated with laser irradiation (λ = 808 nm, 1.0 W/cm^2^) for 1 min. For the evaluation of PTT effect, the cells were costained with calcein-AM and PI (Thermo Fisher Scientific, Waltham, MA, USA) for 30 min. After washing twice with PBS, the stained cells were observed under the fluorescent microscope (Nikon).

### 4.6. HT-29 Xenograft Mouse Model

Animal studies were performed in accordance with the guidelines approved by the Chonnam National University Animal Research Committee (CNU IACUC-H-2023-57). Adult (6-week-old, ≈25 g) male athymic nude mice were purchased from OrientBio (Gwangju, Republic of Korea). HT-29 cancer cells were cultured and suspended in 100 μL of PBS before being subcutaneously inoculated in the right flank of each mouse (1 × 10^6^ cells per mouse). When tumor sizes reached about 1 cm in diameter between 8 to 10 days post-inoculation, IR-783 dissolved in PBS was administered intravenously. Animals were euthanized for in vivo NIR fluorescence imaging within a designated period of time.

### 4.7. In Vivo Time-Dependent Tumor Imaging

In vivo NIR fluorescence imaging was performed using an FOBI imaging system (NeoScience, Deajeon, Republic of Korea). Mice (3 mice per treatment group) were imaged for 48 h after injection to confirm the time-dependent tumor accumulation of IR-783. The fluorescence intensity of the tumor site was analyzed using ImageJ software (National Institutes of Health, Bethesda, MD, USA, https://imagej.net/ij/, accessed on 1 April 2024). Temperature changes at the tumor site were monitored using a thermal imager (FLIR Systems).

### 4.8. In Vivo Photothermal Therapeutic Efficacy

IR-783 or PBS were intravenously injected into the HT-29 tumor-bearing mice (3 mice per treatment group) and the mice were anaesthetized after 24 h. The 808 nm laser with 1.0 W/cm^2^ power density was irradiated on the tumors for 5 min. The thermal imager (FLIR Systems) was used to monitor the temperature changes at the tumor area in real time. At 24 h post-irradiation, the mice in each treatment group were anaesthetized for collecting tumors to observe the histological changes after the H&E staining process. The tumor growth and body weight of mice in each treatment group were observed for 9 days to evaluate the photothermal therapeutic efficacy and systemic toxicity, respectively. The tumor volume (V) was measured by the following formula: V = 0.5 × longest diameter × (shortest diameter)^2^.

### 4.9. Histological Analysis

Resected tumors were preserved for H&E staining and microscopic observation. The tumors were fixed in 4% paraformaldehyde and flash-frozen in an optimal cutting temperature (OCT) compound using liquid nitrogen. Frozen samples were cryosectioned (10 µm thick), stained with H&E, and observed using a microscope. Histological analysis was performed on the Nikon Eclipse Ti-U inverted microscope system. Image acquisition and analysis were performed using the NIS-Elements Basic Research software, https://www.microscope.healthcare.nikon.com/products/software/nis-elements/nis-elements-basic-research, accessed on 1 April 2024.

### 4.10. Statistical Analysis

Statistical analysis was performed by a one-way analysis of variance (ANOVA) followed by Tukey’s multiple comparison test. The results were represented as mean ± standard deviation (S.D.). A value of *p* < 0.05 was considered statistically significant. Curve fitting was performed using the Prism software version 5.01 (GraphPad, San Diego, CA, USA).

## Data Availability

The data are contained within the article.
